# How, when and where? A systematic review on abortion decision making in legally restricted settings in sub-Saharan Africa, Latin America, and the Caribbean

**DOI:** 10.1186/s12905-022-01962-0

**Published:** 2022-10-10

**Authors:** Laura Hinson, Anam M. Bhatti, Meroji Sebany, Suzanne O. Bell, Mara Steinhaus, Claire Twose, Chimaraoke Izugbara

**Affiliations:** 1 International Center for Research on Women, Washington, DC USA; 2 JHPEIGO, Baltimore, MD USA; 3 Save the Children, Washington, DC USA; 4 Department of Population, Family and Reproductive Health, Johns Hopkins Bloomberg School of Public Health, Baltimore, MD USA; 5 WomenStrong International, Washington, DC USA; 6 Welch Medical Library, Johns Hopkins School of Medicine, Baltimore, MD USA

**Keywords:** Abortion decision-making, Systematic review

## Abstract

**Background:**

With increasing global availability of medication abortion drugs, a safer option exists for many women to terminate a pregnancy even in legally restrictive settings. However, more than 22,000 women die each year from unsafe abortion, most often in developing countries where abortion is highly legally restricted. We conducted a systematic review to compile existing evidence regarding factors that influence women’s abortion-related decision making in countries where abortion is highly legally restricted.

**Methods:**

We searched ten databases in two languages (English and Spanish) for relevant literature published between 2000 and 2019 that address women’s decision-making regarding when, where and how to terminate a pregnancy in sub-Saharan African, Latin American and the Caribbean countries where abortion is highly legally restricted.

**Results:**

We identified 46 articles that met the review’s inclusion criteria. We found four primary factors that influenced women’s abortion-related decision-making processes: (1) the role of knowledge, including of laws, methods and sources; (2) the role of safety, including medical, legal and social safety; (3) the role of social networks and the internet, and; (4) cost affordability and convenience.

**Conclusions:**

The choices women make after deciding to terminate a pregnancy are shaped by myriad factors, particularly in contexts where abortion is highly legally restricted. Our review catalogued the predominant influences on these decisions of when, where and how to abort. More research is needed to better understand how these factors work in concert to best meet women’s abortion needs to the full limit of the law and within a harm reduction framework for abortions outside of legal indications.

**Supplementary Information:**

The online version contains supplementary material available at 10.1186/s12905-022-01962-0.

## Background

Globally, approximately 45% of abortions are considered unsafe, defined as a “procedure for terminating an unwanted pregnancy either by persons lacking the necessary skills or in an environment lacking minimal medical standards, or both” [1]. Nearly all of these unsafe abortions (97%) occur in low-resource settings where safe abortion is legally restricted and postabortion care services are limited [2]. However, medication abortion drugs, particularly misoprostol, have become increasingly available in low- and middle-income countries in recent years [3]. Access to and use of medication abortion drugs has dramatically impacted the abortion landscape in legally-restrictive settings and even more permissive settings with limited safe abortion services, presenting a safer option for women who self-manage their abortion outside the formal healthcare system [4–9]. Indeed, informal use of these drugs (e.g. obtaining them without prescription or from a source such as an online seller) in settings where abortion is illegal has been associated with decreased abortion-related complications [10–12]. In addition, because medication abortion drugs expand access to safe abortion care, people are better able to avoid unsafe abortion and its sequelae, exercise their rights, build healthy families and make better decisions about their futures [13].

Despite the availability of medication abortion, even in legally restrictive settings, evidence suggests use remains low [14, 15]. Understanding how women make decisions about what methods and sources to use when seeking to terminate a pregnancy can help to identify points of intervention that can shift women’s choices towards safer termination options, like medication abortion [16]. However, we currently lack a synthesis of the evidence regarding factors influencing women’s decision making around their abortion trajectory and the barriers that restrict some women’s use of safer termination methods and sources – especially in countries where abortion is highly legally restricted. Nearly all studies related to abortion decision making focus on the decision of whether or not to abort [17], and much is already known about individual characteristics and predictors of decision making [18].

Other systematic reviews on abortion in low-and middle-income countries have covered topics such as knowledge, attitudes, and practices among adolescents [19], abortion stigma [20] and abortion and long-term mental health outcomes [21]. These reviews help to shape our understanding of abortion overall but fail to synthesize evidence related to women’s experiences once they determine they will have an abortion. One recent review [22] points to myriad of reasons why women choose informal sector abortions, such as fear of mistreatment by staff, long waiting times, cost, privacy concerns, and insufficient knowledge – but this review is in the context of settings where abortion is legal.

Our review fills this gap by systematically synthesizing the literature related to how, when, and where women terminate their pregnancies and the factors that influence these decisions in legally restrictive settings *after* making the decision to terminate. In this review, we focus on the micro-level aspects of the abortion-seeking process in legally restrictive settings, synthesizing findings from studies exploring women’s individual decision-making processes. The review covers peer-reviewed studies published on countries in sub-Saharan Africa (SSA) and Latin America and the Caribbean (LAC) where abortion was ‘highly legally restricted^1^’ at the time of the study according to the Guttmacher-developed categorization [23]. We choose these geographies because they are under-represented in the literature on abortion-related decision making, because our team members work in these regions and could best interpret the literature, and to keep the scope manageable.

## Methods

### *The Coast* et al *framework for women’s abortion-related care*

We began our work by reviewing a conceptual framework developed by Coast et al. [24] for understanding women's trajectories in seeking abortion care. We found this framework useful as an anchoring point as it is evidence-based and comprehensively incorporates factors that may influence a woman’s trajectory to obtain abortion-related care. In the framework, the authors suggest that abortion-related care for an individual pregnancy includes the interaction of women’s abortion-specific experiences, their individual context, and the regional, national, and international context [24]. Given our aim to understand the individual factors related to women’s experiences making decisions about how, when, and where to abort, we concentrated on the domains of abortion-specific experiences (such as ability to access resources), as well as individual context (such as knowledge, beliefs, and individual characteristics). Throughout our analysis, we returned to this framework to cross-check our findings against the domains and individual components to ensure we were comprehensively capturing all content.

### Search strategy

We searched PubMed, EMBASE, the Cochrane Library (reviews, protocols, and trials), WHO Regional Indexes, Ovid Global Health, JSTOR, POPLINE, CINAHL, and the Web of Science databases for relevant peer-reviewed articles in English and Spanish; we excluded French studies given the language limitations of the team. We restricted our search for articles published from 2000 to correspond with the signing of the United Nations Millennium Declaration—which signified the global community’s commitment to combating poverty and disease and whose indicators directed attention toward the need for safe abortion care—and to limit the review to a manageable twenty-year period that would reflect a contemporary summary of the evidence.

To keep the review narrow in scope, reflect the nature of the Coast et al. [24] framework, and focus explicitly on those whose experiences we sought to understand (i.e. women and girls themselves), we restricted our search to research involving women who had obtained an induced abortion. We included studies on all forms of induced abortion that met the criteria, including legal and illegal and safe and unsafe induced abortions. Studies focusing solely on the decision made regarding whether or not to abort and reasons for abortion were excluded. We also excluded studies on women treated for spontaneous abortion and those focused on the perspectives of providers and/or male partners of women who obtained an induced abortion.

We focused our search on the abortion-related decision-making process for women who obtained an induced abortion. Specifically, we focused on the individual aspects of how, when, and where women choose to induce an abortion. Decision-making for ‘how’ related to who performed the abortion (e.g. the woman herself, a trained clinician, or an unlicensed provider) and the type of procedure (e.g. medication or surgical). Decisions related to ‘when’ focused on the point in time during the pregnancy that women sought and obtained the abortion, or factors that contributed to delays in seeking or obtaining care. Finally, decisions on ‘where’ involved geographic considerations (such as proximity to women’s homes) and the provider/facility type (e.g. private, public, informal healthcare sector). We cross-checked our search terms against relevant components of the Coast et al*.* framework throughout the search process [24]. This review is limited to peer-reviewed articles published in English or Spanish and that focused on the direct experiences of women who report obtaining an induced abortion. The countries included in the study also have vastly differing types of legal restrictions related to abortion. But they share several similar trends related to abortion. Rates for abortion are fairly similar in the two regions, ranging between 36 (Africa) [25] and 44 per 1000 women (Latin America) [26]. In both regions, more than 95% of women of reproductive age live with restrictive abortion laws [23].

We conducted the search in two phases. The first search took place in June 2019 and the second was an update to the review in June 2022, during which time we screened articles published after mid-2019 through mid-2022. While the exact search terms varied by database, the searches all included three components: (1) abortion; (2) women’s experience/decision-making, and; (3) geography. Our final list of search terms is included in Additional file 1: Document 1. We registered the protocol for the systematic review to PROSPERO and reported results using PRISMA guidelines [27].

### Article selection, article quality evaluation and analysis

After removing duplicate search results, we imported all remaining articles into Covidence online systematic review software for title, abstract, and full-text screening [28]. Two reviewers independently screened the title and abstract of each article identified in the search to determine whether the study met the inclusion criteria. Any discrepancy between reviewers was resolved by the full team of reviewers, who jointly made the final decision about whether the paper was included in the full-text review. Once the title and abstract screening was complete, two reviewers independently conducted the full text review of each potentially eligible article. Reviewers again resolved any disagreement over the inclusion of an article through discussion. We evaluated the quality of all included studies using an adaptation of the Critical Appraisal Programme (CASP) quality assessment tool [27]. Results of quality assessment are available on request. Two reviewers independently assessed each article and assigned an overall quality ranking of “low”, “medium”, or “high” quality. Reviewers resolved all discrepancies in these rankings through discussion. We used a standardized form to extract data relevant to the following categories: author names and title; publication and study years; study aim(s); study design; sampling strategy; data collection methods and setting; sample size and characteristics; inclusion and exclusion criteria; analysis methods; and relevant sample for the systematic review. We did not exclude any studies based on the quality assessment. Relying on Thomas and Harden’s [29] thematic synthesis approach, we iteratively and collaboratively abstracted findings into analytical themes. Table 1 [see end of document] highlights the studies included in the review.

**Table 1:** Study characteristics of relevant data* and critical appraisal skills programme (CASP) quality assessment of included studies

References	Country/ countries	Aim	Context/Setting	Study period	Study design	Data collection method (sample size and target population)	Sample characteristics (n) (age; education; relationship status)	CASP Rating
Appiah-Agyekum [47]	Ghana	Highlight the abortion experiences of university students with particular reference to pharmaceutical drugs	University campuses in Ghana	2015	Qualitative	In-depth interviews (*n* = 32 university students)	Aged 18 + years; first -second year (18), third year (8), fourth year (6); single (9), married (4), dating (19)	Medium
Atakro et al. [31]	Ghana	Assess contributing factors to unsafe abortion practices in the Ashanti Region	Four district hospitals in the Ashanti Region of Ghana	2018	Qualitative	Semi structured interviews (*n* = 35 patients)	Patients aged 15–49 years; (5) no education, (15) primary to secondary, (15) postsecondary education; (33) single, (2) divorced	Medium
Baum et al. [43]	Brazil, Nigeria	Explore women’s expectations and experiences with the service they received, the role of stigma and their perceptions of quality care, and how their knowledge and attitudes toward medication abortion shifted in the process	Helplines that provide services to women living in legally restrictive settings	2017	Qualitative	Interviews with women who had been associated with the project helplines (*n* = 30)	Mean age 31; married (*n* = 13), were currently paid for work (*n* = 17), had one or more children (*n* = 16)	Medium
Baxerres et al. [45]	Benin Burkina Faso	Document the means women use to obtain abortions and to learn whether use of misoprostol has become an alternative to other methods of abortion	One health center in Cotonou (Benin); one health center in Ouagadougou (Burkina Faso)	2014–2015	Qualitative	In-depth interviews (*n* = 34) [21 Cotonou (Benin) and 13 Ouagadougou (Burkina Faso)]	(14) aged 18–25 years, (14) aged 26–36 years, (6) aged 37–43 years; Single with no children (14), had children and not living with partner (8), had children, and living with a partner (12)	Medium
Berry-Bibee et al. [68]	Haiti	Learn about illegal abortion access, methods, and perceived barriers to abortion-related care, and to identify the proportion of unscheduled antepartum visits to a public hospital that were attributable to unsafe abortion	University hospital in Cap-Haitien	2013–2014	Mixed methods	Cross sectional survey (*n* = 255 women) and focus group discussion (*n* = 62 women)	Survey participants: (82) aged 18–24 years, (118) aged 25–35 years, (55) aged 35 + years; (70) primary school or less, (161) some secondary school, (24) secondary school or beyond; (155) married/cohabitating, (99) single/divorced/widowed Focus group participants: Mean age: 28 years (20–50 years)	High
Biney and Atiglo [67]	Ghana	Examine the motivations for women’s preference for abortion to resolve unwanted or unintended pregnancy; and investigate the linkages between reasons for abortion and safety of the method employed with a goal to identify which motives warranted safe or unsafe abortion methods	Ghana Maternal Health Survey	2007	Quantitative	Secondary analysis (*n* = 552 women who terminated a pregnancy within the last 5 years)	Age at the time of abortion: (147) aged 15–19 years, (279) aged 20–29, (127) aged 30 + years; (174) none to primary education, (379) secondary or higher	High
Brack et al. [33]	Colombia	Identify the key barriers to legal abortion and to explore the ways they may work separately and together to delay the receipt of high quality, legal abortion care	4 health clinics in Bogota, Colombia	2014	Qualitative	In-depth interview (*n* = 17)	Participant age: (7) aged 18–24 years, (8) aged 25–31 years, (2) aged 32–39 years; (0) primary education, (14) secondary education, (3) university education; (9) single, (8) in a relationship	High
Burkhardt et al. [69]	Democratic Republic of Congo	Examine women’s perception of and access to abortion of sexual violence-related pregnancies in armed conflict context	Bukavu, DRC	2012	Qualitative	In-depth interviews (*n* = 55 women but only 17 women who had an induced abortion)	Participants in parenting and termination group: mean age 33.7 years; (28) no education, (17) any primary school, (7) any secondary school; (16) divorced or separated, (13) widowed, (11) married, (11) single, (4) husband missing	High
Bury et al. [58]	Bolivia	Understand the knowledge and attitudes of women regarding abortion, their responses to an unwanted pregnancy, and what they experience when they seek to induce abortion	Peri-urban areas of Sucre, Santa Cruz, Cochabamba, La Paz, and El Alto	2010	Mixed-Methods	Focus group discussion (*n* = 115 women) In-depth interviews (*n* = 50 women)	Not available for FGD and IDI participants	Medium
Byrne et al. [65]	Nigeria	Examine factors that influence women’s decisions about where to terminate their pregnancy and their corresponding preferences regarding abortion provider/location. Examine the reasons that hindered women’s ability to operationalize their preference and to compare characteristics of women who were able and unable to use their preferred abortion provider	Six states (Anambra, Kaduna, Lagos, Nasarawa, Rivers, and Taraba)	2018 and 2019–2020	Quantitative	*n* = 1144 women who reported having done something to remove a pregnancy or bring back a period when they were worried they were pregnant in the 2018 survey	At the time of their abortion, most women were under the age of 30 years and at peak childbearing: 2.8% were under 15 years, 18.3% were 15–19 years, 29.2% were 20–24 years, and 22.0% were 25–29 years. Over half of women were married (53.0%) 28.8% were attending school, 48.6% had children, and 69.0% were living in an urban setting	High
Chareka et al. [49]	Zimbabwe	Understand the economic and social influences on abortion decision-making behavior and abortion practices amongst a group of young women who sell sex	Urban and peri-urban areas of Harare and Bulawayo	2019	Qualitative	42 IDIs with young women who sell sex (some of whom had had an induced abortion)	Not available for IDI participants	Medium
Cleeve et al. [53]	Uganda	Explore reproductive agency in relation to unsafe abortion among young women seeking post abortion care	Mulago Hospital in Kampala, Uganda	2013	Qualitative	In-depth interviews (*n* = 18 women)	17 participants: (7) aged 16–18 years, (6) aged 19–20 years, (4) aged 21–25 years; (4) primary education, (11) secondary education, (2) missing; (16) unmarried, (1) married	Medium
DePiñeres et al. [34]	Colombia	Examine the delays and barriers among women denied abortion care; and explore the factors that enabled or prevented women from seeking safe and legal services after being denied care and whether women used or considered using informal sector abortion methods outside the formal health system after denial	Fundación Oriéntame in Bogota, Colombia	2013	Qualitative	In-depth interviews round one (*n* = 21 women) Round two (*n* = 8 women)	Most participants were aged 19–24, (3) aged 16–17 years	High
Domingos et al. [72]	Brazil	Understand the experience of women who induced an abortion during adolescence as demanded by their mothers	Minas Gerais, Brazil	2010	Qualitative	In-depth interviews (*n* = 3 women)	Aged 18–27 years, all women were single and did not finish high school	Low
Esia et al. [55]	Ghana	Examine the pre and post abortion experiences among young women	Planned Parenthood Association of Ghana	2011	Qualitative	In-depth interviews (*n* = 21 women)	(14) aged 11–16 years, (7) aged 17–21 years, (1) no education, (9) junior high school, (8) senior high school, (1) tertiary education, (6) completed education	Low
Ferrari et al. [50]	Brazil	Critique of the invisibility of illegal abortions among adolescents and discuss the specificities of the practice in this life cycle stage	South Zone of Rio de Janeiro	Not stated	Qualitative	Ten in-depth interviews with young girls ages 15 to 17 who had undergone an abortion between 12 and 17	Five reported “no religion.” Nine declared themselves to be “black” or “brown” and only one declared herself to be “white”. All adolescents lived with their mothers, who became pregnant during adolescence or early adulthood, between the ages of 15 and 24. Three adolescents (1, 2, 3) did not live with their fathers and four (1, 2, 3 and 6) also lived with their grandmothers.” Of the participants, nine were students at the time of the abortion, and they were attending between the 7th and 11th grades, which was compatible with their ages. Only one adolescent (10) was not a student, due to having started working as a salesperson after her first pregnancy, at age 15, which she carried to term	Medium
Frederico et al. [61]	Mozambique	Explore the individual, interpersonal and environmental factors behind the abortion decision-making process	Maputo and Quelimane	2016–2017	Qualitative	In-depth interviews (*n* = 14 women) *n* = 9 women in Maputo *n* = 5 women in Quelimane	Median age 21; (4) primary education, (8) secondary education, (2) university	Medium
Freeman [52]	Chile Peru	Use the concept of biopolitics to analyze how the governing of women’s bodies forces them into certain spatial movements namely the crossing of international borders for abortion services	Arica, Chile and Tacna, Peru	2013	Qualitative	In-depth interviews (*n* = 1 woman)	Not described	Low
Hess [70]	Gabon	Explore the reasons women chose to terminate their pregnancies, the methods used to induce abortions and post abortion effects experienced by these women	Hospital in Ngounie province	2002	Qualitative	In-depth interviews (*n* = 5 women)	Aged 18–34 years; (3) single and (2) married	Low
Hill [35]	Ghana	Explore the context and methods used in unsafe abortions with the aim of identifying areas that governments must consider when operationalizing their strategies to reduce deaths from unsafe abortions	Kintampo, Ghana	2005	Qualitative	Focus group discussions (*n* = 8 groups with women)	Not described	Low
Izugbara et al. [36]	Kenya	Address the knowledge gap regarding the social dimensions of abortion safety	Health facilities providing post abortion care in Kenya	2012–2013	Qualitative	In-depth interviews (*n* = 50 women treated for complications due to unsafe abortions)	(12) Aged 18 or less years, (24) aged 19–24 years, (11) aged 25–30 years, (1) aged 31–34 years, (2) aged 35 + years; (25) primary education, (13) secondary education, (12) tertiary education; (32) never married, (12) married, (5) separated/divorced/deserted, (1) widowed	High
Juarez et al. [32]	Mexico	Explore women’s experiences of induced abortion in three federal entities with very different legal contexts, and investigate if abortion-seeking patterns and experiences differ across these settings	Three states: Queretaro, Tabasco and the State of Mexico (to compare three degrees of restrictiveness of abortion legislation)	2014–2015	Qualitative	Semi-structured interviews (*n* = 60) among women 18–44 who were low to middle income	Most women were between the ages of 25 and 29 (*n* = 18). Half were single and half were married. The majority had finished high school (*n* = 25). Nineteen women had zero children and 27 had two or more	High
Katz et al. [62]	Nigeria	Examine how abortion clients from a range of care models in Nigeria perceive abortion and explore the role their beliefs and fears play in their care-seeking experiences and interactions with providers	Lagos and Ogun states from two healthcare clinics a safe abortion hotline and four proprietary and patent medicine vendors	2018–2019	Qualitative	Semi-structured, in-depth interviews (part of a larger study in four countries) (*n* = 25)	The mean age of participants was 25 years, with a range of 16–41 years. Half of the participants had one or more children and nearly two-thirds were unmarried	Medium
Kebede et al. [51]	Ethiopia	Explored abortion-seeking safety considerations in social and cultural detail by exploring the routes to abortion undertaken by young, unmarried women in particular, whose social situation with regard to abortion differs significantly from that of married women with unwanted pregnancies	Addis Ababa	2006–2007 2009–2010	Qualitative	Interviews: (*n* = 25 women)	Age: 18 (20 years or younger), 2 housemaids with primary education, 4 day laborers with 2–3 years primary education, 3 students in tertiary institutes, 6 high school students, 2 junior high students and waitresses, 3 4–5 years of schools and taking vocational training, 1 assistant cook without formal education, 4 high school graduates looking for a job	High
Keefe-Oates et al. [37]	Colombia	Aimed to gain a deeper understanding of women’s concerns before arriving to abortion careas well as describe women’s experiences receiving support for those concerns during their abortion service, particularly through abortion counselling	International Planned Parenthood/Western Hemisphere Region (IPPF/WHR): Mexfam in Mexico and Profamilia in Colombia	2015–2016	Qualitative	In-depth interviews (*n* = 30 women of which 15 were from Colombia)	Colombian women age 19—38 years	High
Lafaurie et al. [41]	Mexico Colombia Ecuador Peru	Collect information about women’s experiences using misoprostol or methotrexate plus misoprostol under clinical supervision	Clinics	2003–2004	Qualitative	In-depth Interviews with women: urban setting in Colombia (*n* = 11) urban (*n* = 9) and rural (*n* = 6) setting in Mexico urban setting in Peru (*n* = 12) urban coastal (*n* = 5) urban Andean (*n* = 6) setting in Ecuador	Aged 18–44 years; Primary education (17), secondary/professional (32); Employed (28), In School (18); Living with a partner (18); Has children (22)	High
Larrea et al. [64]	Chile	Understand abortion trajectories leading people to use the service called Women Help Women (WHW), their experiences with self-managed abortion, how they evaluate WHW’s quality of care and how they compare it with gynecological and obstetric services they have accessed in the past	Santiago, who had used the WHW service	2019	Qualitative	Seven phone-based and four in-person (*n* = 11)	Age ranges from 23 to 36; majority employed and in a relationship with university degrees	Low
Loi et al. [30]	Kenya	Explore decision-making preceding induced abortion among women with unwanted pregnancies	Jaramogi Oginga Odinga Teaching Hospital and Kisumu East District Hospital/ Kisumu, Western Kenya	2014	Qualitative	In-depth interview (*n* = 15 with 9 women) 7 women from JOOTRH; 2 women from KDH	19–32 years old; Occupation: (3) students; (4) employed; (2) unemployed; Relationship status: (3) partnered; (1) engaged; (2) married; (3) no relationship)	High
Makleff et al., [63]	Kenya	Examine the experiences of women who obtained an abortion with regard to stigma, expectations, and perceptions of abortion quality of care	Thika and Eldoret	2017	Qualitative	Semi-structured interviews (*n* = 34 with 24 in Kenya)	Mean age in Kenya sample was 26.9; majority (*n* = 14) had higher level education, were unmarried (*n* = 15) and had no children (*n* = 11)	Low
Manriquez et al. [46]	Chile	Document the experience of clandestine use of medical abortion among university students	10 universities in Santiago Metropolitan Region	2006–2016	Qualitative	In-depth interview (*n* = 30 women)	Aged 17–26 years; Relationship status: (30) single	Medium
Mitchell et al. [59]	Mozambique	Understand how women evaluate method choices and experiences; how individual women choose whether or not to pursue a home-based abortion method and;how might their perspectives vary based on their personal circumstances, prior experiences, the quality of care rendered, and the clinical outcome	Urban Mozambique	2005–2006	Mixed Methods	In-depth interviews (*n* = 70 women) Clinical survey (*n* = 837)	For IDIs: Mean age 25.6; 56.3%student, 98.9% literate For Survey: Mean ag25.7; 56.8% students; 98.3% literate	High
Mohamed et al. [60]	Kenya	Understand young Kenyan women’s experiences with induced abortion and PAC services.	Marie Stopes clinics	Not stated	Qualitative	In-depth interviews (*n* = 15 women)	(2) Aged 18–19 years, (13) 20–24 years; Marital status: (6) single, (9) In a relationship	High
Omideyi et al. [71]	Nigeria	Explore whether abortion options were chosen and how they were perceived in relation to preventing unplanned pregnancies and births	Osun state in southwest Nigeria	Not stated	Qualitative	In-depth interviews (*n* = 10 women) Focus group discussion (*n* = 3 women)	The mean ages of participants were 24.7 years for women (both IDIs and FDGs). Characteristics of IDI participants: Women: 1 primary school, 1 university school graduate, 2 polytechnic student, 2 university student, 4 secondary school; Marital Status: Females: 4 Married, 6 unmarried	High
Osur et al. [73]	Kenya	Understand the role that social networks play in facilitating access to unsafe abortions done clandestinely at community level	Six health facilities in three districts of Siaya County in the southwestern part of Kenya	2011–2012	Mixed methods	Survey (*n* = 320 women) Case studies (*n* = 2 women)	(110) aged below the age of 18 years and another (133) aged 18–24 years	Medium
Ouedraogo and Sundby [44]	Burkina Faso	Demonstrate how social determinants such as personal resources may determine the type of clandestine abortion women are likely to access (safe or unsafe) and the time taken from knowing that a woman is pregnant to the situation of effective abortion within a country where the access to induced abortion is restricted	Health facilities in Ouagadougou, Burkina Faso	2011–2012	Qualitative	Case studies (*n *= 2 women)	26-year-old unmarried woman, law student; a 25-year-old unmarried woman with 2 children	Medium
Oyefara [74]	Nigeria	Investigated the effects of gender and power relations on decision-making regarding induced abortion among undergraduate students	University in Lagos, Nigeria	2014	Qualitative	In-depth interviews (*n* = 40) (*n* = 24 women)	Characteristics of female participants: (13) aged 20–24 years, (11) aged 25–30 years; marital status (21) single, (3) married	Low
Oyeniran et al. [42]	Nigeria	Assess women’s knowledge; their expectation and experiences of the methods employed for abortion; and their health care-seeking decisions following a complicated abortion	Nine health facilities in the south-west geo-political zone of Nigeria	2013–2014	Qualitative	In-depth interviews (*n* = 31 women)	(58.1%) aged 21 to 30 years, (29.1%) were students	High
Penfold et al. [56]	Kenya	Explore the decision-making, experiences and preferences of women who attended private clinics for safe abortion services	Nine private clinics in Western Region, Kenya	2016	Qualitative	In-depth interviews (*n* = 22 women)	22 women who were most commonly in a relationship or married, aged 23–26 years and had children	Medium
Petracci et al. [54]	Argentina	Provide data and reflections on trajectories and scenes lived by women and men in connection with the decision to terminate a pregnancy and the abortion experience	Buenos Aires, Argentina	2007–2008	Qualitative	In-depth interviews (*n* = 30 women)	10 women aged 18–27 years, 10 women aged 40–49 years; 5 women aged 40–49 years old	Medium
Ramos et al. [57]	Argentina	Explore the experiences of women with the use of misoprostol for inducing an abortion	Public hospital in Buenos Aires metropolitan area	2011–2012	Qualitative	In-depth interviews (*n* = 45 women)	45 women interviewed were aged 18–40 years; 60% had completed basic education	High
Rominski et al. [38]	Ghana	Understand why young women seeking an abortion in a legally enabling environment chose to do this outside the formal healthcare system	3 hospitals	Not stated	Qualitative	Focus group discussions (*n* = 8 groups with 29 women)	aged 13–35 years, with a mean age of 25.7 years	Medium
Seid et al. [39]	Ethiopia	Assess the barriers to accessing safe abortion services from the clients’, health extension workers’ and service providers’ perspective	3 health facilities in Adama and Asella towns in Oromia Region	Not stated	Qualitative	In-depth interviews (*n* = 38 women)	(8) were below 20 years of age, (24) aged 20–30 years; (20) completed primary school, (7) illiterate; (22) married	High
Solheim et al. [66]	Tanzania	Understand how misoprostol is perceived, accessed and used on the ground as well as explore how the use of misoprostol as an abortion drug is shaped by and in turn shape local practices and social relations	Three urban and suburban areas in Dar es Salaam	2015	Qualitative	In-depth interviews with young women who had used drugs to induce an abortion (*n* = 15)	Not presented for IDI participants	High
Szulik and Zamberlin [75]	Argentina	Understand the stigmatizing experiences of women related to abortion	Buenos Aires (city and province) and Chubut province	2016	Qualitative	In-depth interviews with 18 women	Not presented	Medium
Szwarc and Vásquez [48]	Argentina	The study set out to investigate the waiting and the temporalities during the abortion process in women's experiences residents in the Metropolitan Area of Buenos Aires	Buenos Aires metropolitan area	2015	Qualitative	In-depth interviews (*n* = 5 women)	Aged 26–36 years All attended or completed a tertiary education or university	Medium
Yegon et al. [81]	Kenya	Understand the link between abortion-related stigma and unsafe abortion from the perspective of women seeking induced abortion services or post abortion care (PAC) in health facilities	Machakos County in Eastern Region and Trans Nzoia County in Rift Valley Region	2014	Qualitative	In-depth interviews (*n* = 26 women)	6 of those that sought PAC were aged above 25 while 5 were aged below 25; 9 aged above 25 years and 6 aged below 25 years of those that sought induced abortion	High

*Relevant data indicates data that were analyzed within this systematic review's scope, which includes only study populations of women who had an inducted abortion, in included countries, etc

We uploaded included articles into Nvivo and coded them using a codebook based on the Coast et al. framework. We double coded approximately 20% of articles for inter coder reliability, resolving all discrepancies in coding and finalizing the codebook. We coded relevant sections of the articles, mainly in the results sections, pertaining to findings that fit within our inclusion criteria. This meant that we included any findings related to women who had an induced abortion in the included countries/regions. Content from countries or regions outside our scope, from additional perspectives beyond women who had induced abortions, or not related to the decision-making process around when, where and how to abort was excluded.

## Results

The initial Phase 1 search conducted in 2019 yielded 11,620 articles. After removing duplicates in EndNote, we imported 6787 articles into Covidence, which identified a further 17 duplicates, and screened the titles and abstracts of 6770 unique articles. We identified 95 potentially relevant articles and retrieved the full text manuscript for further evaluation. After completing full text reviews, we excluded 59 articles from the sample, leaving 36 articles that met study inclusion criteria. The Phase 2 search began with 2043 articles, of which 1874 were imported into Covidence for title and abstract screening, after duplications were removed. We identified 23 potentially relevant articles and reviewed the full text of each manuscript; of these, ten were included.

**Fig. 1:**
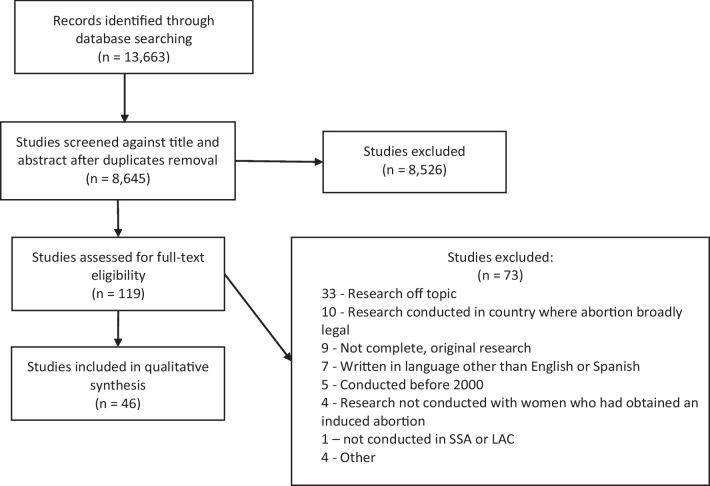
PRISMA Flow diagram of study inclusion process

See Fig. 1 for the flow diagram of the search.

Of the 46 studies included in the systematic review, 40 were qualitative, four employed a mix of quantitative and qualitative methods, and two were exclusively quantitative. Our analysis synthesized findings from primary research conducted across 21 countries: twelve countries in sub-Saharan Africa and nine in Latin America and the Caribbean. The studies varied greatly with regard to methodological rigor and depth of analysis. Most studies (*n* = 20) met the high-quality rating on our CASP quality assessment tool while 18 articles were rated medium quality and eight were rated low quality. The characteristics of the 46 studies included in our synthesis are reported in Table 1, along with their CASP assessment rating.

### Thematic findings

The findings of this review are organized across four broad themes: (1) the role of knowledge; (2) the role of safety; (3) role of social networks and the internet, and; (4) cost, affordability and convenience. In addition to direct quotes from participants cited in the reviewed studies, we use quotes from authors that summarize findings from their own research.

### The role of knowledge

The role of knowledge factored prominently into women’s decisions regarding how, when, and where to terminate their pregnancy. This included aspects broadly related to knowledge of the abortion law and knowledge of methods and sources of abortion. These two categories of knowledge acted upon women’s abortion-related decisions directly and indirectly as described below.

#### Knowledge of law

Knowledge of abortion laws affected women’s abortion-related decision-making by impacting their perceived or actual choices [30–40]. The impact of one’s knowledge of the abortion law varied by what those views entailed. Women’s understanding that abortion was broadly illegal, regardless of whether their knowledge was accurate, was associated with fear of potential legal or other repercussions of seeking care at a formal health facility. Thus, perceptions of illegality restricted women’s choices were linked to use of less medically safe (as distinct from socially or legally safe) abortion methods and sources [31–33, 36, 37]. This perception is illustrated by one woman in Atkaro et al. [31] *I knew that it is illegal to have an abortion in Ghana and so I could not have gone to any facility to have by pregnancy terminated. All my friends that I asked only recommended some herbal mixture called agbeve for me… Although I know I could bleed to death from terminating my own pregnancy, I didn’t have a choice or options. So, I used the agbeve herbal mixture*.

Many women who were unsure of the law or who had anxiety about whether their situation qualified as a legal indication tended to seek abortion outside the formal health sector [31–33, 36, 37]. As Izugbara et al. [36] summarized: *Respondents generally believed that abortion is illegal in Kenya, mentioning the Kenyan media, religious leaders, health providers, family, friends, and schools as sources of their information on the criminality of abortion. Given the presumed illegality of abortion in Kenya, safe abortion was also understood in terms of procedures and providers that shielded women from the law and arrest.*

As such, not knowing the legality of abortion or the exceptions in the law presented barriers to timely care [33]. Conversely, learning about conditions under which women could seek or be eligible for legal abortion services tended to positively impact women’s abortion-related decision-making [32, 34, 37], for example, giving participants more confidence to advocate for themselves and their desires, and empowered to make well-informed decisions [34]. The process of learning about available legal services was often facilitated by an advocacy group or legitimate service provider [33].

#### Knowledge of methods and sources

Women’s knowledge of specific abortion methods and sources was a proximal factor that directly impacted their abortion-related decisions. Lacking awareness of methods and sources was common [30, 31, 33, 34, 39, 41–48] and was an obstacle to women using a medically safe method or source in one of two ways: either women would act upon the limited knowledge they had, which tended to lead them to obtain an unsafe abortion; [31, 39, 46] or their lack of knowledge led to delays, which in effect limited their options as a result of later gestational age [33, 34]. Regarding the latter experience, Seid et al. [39] summarized findings as such: *Lack of information and knowledge about safe abortion services is the barrier. If they (women) do not have information, they hesitate to decide and as time goes, they do not have the chance to terminate their pregnancy. The only option they have may be giving birth.*

Whether a woman had or could access information about safe abortion methods and sources was often related to her demographics. Rural, older, and less educated women, as well as those with less social capital (namely not having medical providers in their social network) were unlikely to have adequate information to make an informed decision and use a safe method [39, 47–49] While many women are aware of both safe and unsafe methods or sources, knowledge of misoprostol and mifepristone specifically appeared more common among younger women [48]. In one Kenyan study, information about abortions came from informal social networks from high school and from friends with a prior abortion experience [30, 50].

A lack of knowledge about methods and sources was not necessarily linked with a preference for a certain type of method; in fact, incomplete information led to significant misperceptions and heterogeneity in preferences [42]. It also resulted in incorrect use of medical abortifacients and concerns about product effectiveness. In some cases, this contributed to women preferring surgical abortion while for others, it led to a preference for medical abortion.

In societies where abortion is highly stigmatized, women tend to lack access to information about safe abortion methods or where they can be procured [51, 52]. As Kebede et al*.* [51] points out: ‘*all [women in the study] struggled to access information about abortion possibilities and attributed this difficulty to the morally charged silence surrounding abortion and premarital sexual activity.*’ Even in countries with more permissive laws, participants were often unaware of them because of the shroud of taboo [34].

#### The role of safety

In addition to women’s knowledge, their perceptions of medical, legal, and social safety were significant factors in their decision making regarding how and where to terminate their pregnancy [31, 33, 36–42, 44, 47, 48, 51–61]. While women strongly preferred their abortions to be medically safe, concerns about legal and social safety often prevailed, leading women to have a medically unsafe termination.

#### Perceptions of medical safety and quality

In the absence of other influencing factors, women strongly preferred medically safe abortion methods and services [37, 48, 52, 55, 56, 62]. Based on a study in Ghana, Esia et al. [55] summarized that ‘*All the respondents indicated that they preferred to have abortion at recognized facilities and by recognized practitioners so as to make it safe*’ However, there was significant variation in what methods women perceived as most safe. One study found that women perceived abortions induced by ingesting substances to be safer than surgical abortions because they associated surgical equipment with a greater risk of complications like infections [51]. In other cases, women stated preferences specifically for medical abortion due to the perception of lower health risks [48, 58, 63]. Regardless of preferred method or source, women’s preferences for medically safe abortions were often overridden by greater concerns about legal and social safety.

Women’s perceptions of the quality of care provided at facilities played a role in the decision-making process regarding where to seek abortion services [36, 37, 43, 44, 47, 51, 55–57, 59, 62] These perceptions included likelihood of respectful care and willingness of the provider to perform the requested procedure [33, 34, 57]. In Nigeria, perception of care had more to do with having a good reputation, i.e. not being a “quack [43, 62]”: “*They noted that individuals who seek care from so-called “quacks” suffered from side effects and “regret it,” but for those who obtain services from a qualified provider “there won’t be any problem* [62]*.”*

Respectful care was generally identified as provider(s) having the interpersonal skills necessary to treat women with unwanted pregnancies – regardless of sociodemographic or marital status – with empathy and respect [33, 34]. Two studies found that women expected to experience disrespectful care (such as manipulating women to carry to full term or belittling a woman’s decision to abort) at public health facilities, leading them to instead choose facilities or providers recommended by friends [40, 57].

#### Perceptions of legal safety

Fear of legal repercussions often superseded women’s preferences for medically safe abortion methods and services, leading them to attempt to self-induce using unsafe methods and/or seek care from clandestine providers [31, 37, 38, 40, 49, 64]. In other instances this led women to withhold information from postabortion care (PAC) providers about their previous attempts to self-induce [40, 48, 58]. As Rominski, Lori, and Morhe [38] found: *The legal status of abortion was mentioned by all groups of participants as a reason for why women self-induce rather than come to facilities for abortion services. Prosecution of women, or their providers, due to inducing an abortion is rare, but women are afraid of this potentiality.*

As reported by Manriquez et al*.* [46]. women often lie to PAC providers about their attempts to self-induce for fear of legal consequences. This is in accordance with advice from harm reduction information handbooks, which enabled women to receive treatment while ‘reducing the risk of rejection and denunciation’ [48]. In interactions with providers, these investigators observed that ‘*None of these women mentioned they had induced an abortion. They had all decided not to tell in advance. To ensure they succeeded in this they kept silent, denied it, lied, accepted rough treatment, did not express any pain, and did not ask for information.*’ [48].

Even if providers had their suspicions, women’s fears of legal punishment often led them not to reveal prior attempts to self-induce or receipt of induced abortion care from clandestine providers [38, 46].

#### Perceptions of social safety

Of all three types of safety concerns—medical, legal, and social—concerns for social safety had the greatest influence on women’s decisions regarding how and where to terminate their pregnancy [33, 36, 38, 39, 41, 43, 44, 47, 48, 51, 53, 56, 58–62, 65]. Social safety encompasses abortion providers’ and others’ ability to maintain the secrecy of a woman’s abortion experience. Fear of stigma or social repercussions influenced women’s decisions about which method to use, but not consistently towards or away from any particular method [48, 53, 58, 59, 62, 66]. Women who preferred medical abortion cited the increased privacy possible through minimizing the number of hours spent in a hospital, as well as the risk of being recognized by or experiencing unwanted attention from others at or near the facility [38, 41, 44, 48, 58, 59, 64–66]. As one participant reported from Chile: “*I am grateful that I was able to do this (abortion) quietly, alone in my home, and not with doctor* [64].” However, the same motivation for privacy led other women to select other methods or sources [36, 38, 44, 56, 59]. Women who preferred surgical abortion appreciated a sense of privacy from fewer visits in comparison to medical abortion, which women believed may involve multiple visits in the case of excessive bleeding, which they perceived as a common side effect [44, 56, 59].

Fear of stigma or social repercussions caused many women—particularly young, unmarried women—to choose riskier methods or services in order to reduce the social risks [33, 38, 41, 47, 51]. Young women were likely to ingest harmful substances and/or avoid formal healthcare settings initially because of a desire to keep the abortion private and avoid involving their parents [47, 51, 53, 60] or social network [39, 47, 51, 53, 60, 61]. The perceived lack of confidentiality in high-profile health facilities led women to prefer to terminate elsewhere even when the high-profile facilities were thought to have the best equipment and providers [36, 51]. These concerns were particularly acute for young girls, who feared that these more legitimate facilities may contact their parents or guardians [41, 59].

Finally, fear of stigma or social repercussions led some women to choose services distant from their home [41, 42, 47, 48, 51, 52], and to choose discrete albeit unsafe methods and places to terminate the pregnancy [51, 53, 58, 60]. As Mohamed et al*.*[60] found: ‘*In addition to strong religious and cultural beliefs preventing women from seeking out abortion services at healthcare facilities, many communities also use stigma, isolation and shame as tools to ensure that women do not break from tradition.*’

#### Role of social networks and the internet

Social networks mainly influenced women’s decision regarding how and where to terminate a pregnancy through sharing of information and experiences [30–33, 35, 36, 38, 40, 41, 43, 45, 47, 50, 59, 61, 62, 64, 65, 67–73]. There was no uniform narrative about who a woman tells, gets information from, or involves in her decision-making process. Women most often involved their friends [32, 35–37, 40, 41, 43, 46–48, 50, 55, 58, 62, 65, 70, 71, 73], partners [30, 31, 34–38, 41, 46, 48, 54, 64, 67, 68, 70, 71, 74], and/or family [32, 35, 36, 48, 52, 61, 62, 70, 72, 74], in the decisions related to how, when, and where to abort. Health providers [32, 61, 68] and strangers or acquaintances [43], neighbors [32, 62], “feminist activists [64]” or other NGO staff [32] and abortion ‘brokers’ [45, 51] were also consulted, but with less frequency.

These articles suggested that social networks have significant influence over whether a woman ultimately has a safe or unsafe abortion by affecting her perceptions of methods and sources and their corresponding social and medical safety. In some cases, friends led women to have safe abortions [32, 33, 45, 47, 62], but in many cases they recommended unsafe options [31, 46, 47, 51, 70, 71]. The information and support that women received from friends was often related to her and her network’s social standing. Women of higher socioeconomic status and education, as well as those with connections to people in academic and health sectors, were more likely to experience safe abortion [33, 47, 54, 61, 62, 67], whereas women from social networks who lacked information or connections to knowledge or knowledgeable people tended to experience unsafe abortions [31, 36, 70, 71]. Regardless of the actual method or service they chose, women perceived that their choices were safer if they relied on information from trusted friends [32, 36, 37, 40, 41, 47, 48, 55, 57, 62, 70] or people who had previously successfully aborted [30, 38, 42, 48, 62, 64, 73]. In many cases, involving family members resulted in less safe abortions, especially when it involved unskilled family members as the providers of the abortion method [35, 36, 56, 61, 72]. In contrast, one article suggested that a lack of involvement of any friend or family member in the decision-making process, whether a result of preference or social isolation, led to less safe abortions [51].

When male partners were involved, they were typically most instrumental during the procurement phase—e.g. going as a surrogate to a chemist or pharmacy, identifying a facility-based provider, or financing the procedure [30, 31, 35, 36, 38, 41, 44, 46, 48, 52, 54, 67, 70, 74] In many of these instances, male partners also made the decisions about method and source on behalf of the woman; however, it was not always clear whether this was a situation preferred by the woman or whether she would have liked to be involved in the decision-making.

Women who sought an induced abortion sometimes (but not always) used the internet for abortion decision-making. In some cases, the internet was the main source of information for determining where and how to abort, especially for determining legality of abortion in different states and/or how to get pills [32, 64, 75]. In other cases, women with strong networks did not use the internet or support services to navigate the abortion-seeking process: *“[In Chile], Most participants did not contact [Women Help Women] WHW during the abortion process because they did not need more information, or because they had support from other feminist organizations, acquaintances who had had abortions and trusted health professionals, with whom they could communicate via instant phone messaging* [64]*.”*

#### Cost, affordability and convenience

Evidence suggests that the perceived cost and affordability of specific services often influenced women’s decision-making related to abortion care seeking [30, 34, 36, 40, 41, 51–53, 55, 61, 65]. Some women’s knowledge of safe methods and sources was high, but barriers such as cost and affordability prevented utilization of those methods and sources [30, 36, 49, 51, 53], with perceptions of service affordability linked to women’s economic status and ability to pay [34, 59]. The most obvious way that cost and affordability impacted decision making was regarding whether to get services in public or private facilities. Women with more limited financial resources opted almost unanimously to patronize public clinics or other non-clinical providers in contrast to wealthier women who were more likely to seek services at private facilities [40, 53, 55, 65]. Further, women who were financially better off could procure pregnancy tests earlier following suspicion of pregnancy, which meant the gestational age at which they were making decisions regarding how, when, and where to terminate was earlier compared to women who were economically disadvantaged [54]. As such, because access to early care skewed towards wealthier women, less financially secure women were confronted with greater challenges and vulnerabilities as a consequence of delayed care seeking [51, 53].

Cost and affordability also impacted decision-making on which method—surgical or medical—women chose for their abortion. In general, if women could afford it, they wanted a method that they perceived as quick, efficient, and as painless as possible [44]. Some women viewed a surgical procedure conducted by a doctor as the best option, while others viewed going to a chemist for a medical procedure as best [30, 44, 49]. As reported by Loi et al*.*[30] *Some women [i.e. participants] stated they knew about Marie Stopes, a reliable abortion provider; however, due to high transportation fees they opted for medical abortions using Misoprostol, which was provided by chemists.*

Thus, poorer women were more likely to be constrained in their ability to operationalize their abortion preferences. In some situations, such as in Chile, affordability was a main reason for why women chose to use certain services, including abortion access organizations like Women Help Wome [64]. However, a few studies did not find that cost or affordability were predominant factors in women’s decisions related to when, where, and how they terminated [47, 56, 57, 66].

Finally, several studies showed that decisions about where and how to abort were related to convenience, including factors such as distance to the provider [32, 65], time spent waiting on medical abortion (i.e. pill) shipments [64], or simply a lack of other options: As one Nigerian participant stated, *“The reason I came to [clinic name] is because I do not have any alternative”* (Age 41, clinic) [62].

## Discussion

Our systematic review findings illustrate there are many factors that influence the decision-making process of women obtaining an abortion in highly restrictive legal settings. These results resonate with the Coast et al. conceptual framework of explanatory factors influencing women’s abortion trajectories and, like the framework suggests, highlight how these factors are interrelated and dynamic [24].

Women’s perceptions of abortion method/source safety matter in terms of how, when, and where they induce. Concerns such as fears of legal repercussions and social stigma often supersede preferences for medical safety in these contexts. The ability to maintain discretion and keep the abortion secret is a significant factor motivating many women’s abortion-related decisions; women seek an abortion far from home, in low-quality facilities, or use potentially unsafe methods to minimize the likelihood of being seen by or having their personal information shared with family, friends, or community members. The importance of safety, especially as it relates to discretion, has been shown in other literature as well [20, 76, 77] including for women seeking informal sector abortions in legally permissive settings [22]. This review adds further context of how these perceptions continue to complexly influence those individuals who have already decided to abort. It also extends scholarly understanding of the intersections of women’s abortion care-seeking choices with national legal contexts, highlighting the combination of factors that weigh on women’s decisions as they negotiate access to services.

We found that knowledge of laws, sources, and methods play an important role in women’s abortion decisions related to how, when and where to abort. Similar to what has been found elsewhere, women resort to making choices that are less medically safe where knowledge is limited and abortion is highly stigmatized [22, 76, 78]. Conversely, when women access accurate information even in contexts with highly restrictive abortion laws, they make safer choices and can act more quickly, reducing delays and potential for complications associated with later termination.

The social environment plays a critical role in women’s decision-making. Social networks are key, and women’s decision-making processes and resultant experience of safety is related to the information and resources in their network. Other studies have similarly found that social networks can help women determine how to obtain a clandestine and safe abortion [22, 79] and drive them to a safe abortion experience [76]. Demographics are also related to one’s social network, which has direct implications for the types and quality of information and resources women receive from people within those networks. One’s socioeconomic characteristics can narrow available options, either directly through cost and logistical barriers or indirectly through a less connected and knowledgeable social network and decrease the likelihood that those options involve a medically safe abortion experience. We also found that where social norms dictate that abortion is highly stigmatized, women know little about the abortion laws, have less social support, and thus have fewer viable options regarding where and how to terminate a pregnancy [20].

Our findings speak to the interrelatedness of these three domains, a central aspect of the Coast et al. framework [24]. Women with inaccurate or incomplete information, as well as those who are delayed in learning their pregnancy status and/or with limited social connectivity or support delay care-seeking and have fewer options available to them. While similar findings are echoed in more legally permission settings [22, 76], highly restrictive settings such as those seen in this review may further stigmatize, limit and negatively shape women’s choices and the consequences of those choices.

To support women’s decision-making and their use of safer methods, we must consider how women’s individual perceptions – of care, cost, and safety in the broader sense–shape their choices, and are shaped by their environment, including their social networks.

Policy and programmatic interventions that emphasize and maintain confidentiality are a high priority for women and providers alike, as are social and behavior change interventions that provide women with information about legal exceptions and where they can obtain safe abortion methods. Even in highly restrictive settings, policies, resources, information and counseling services focused on strengthening women’s knowledge of legal indications and supporting them to self-manage their abortion could be made more broadly available [80, 81]. Policies that promote access to quality and timely reproductive health knowledge will ensure that women in all settings have the requisite information to make life-saving decisions that are within legal limits, but that also do not compromise their health. Abortion policy interventions need not only to connect to legal stipulations, but also to what is happening on the ground and to the lived realities of women. Program and policies that support women’s resort to health promoting social networks will save lives and improve long-term wellbeing. Sustained evidence-informed policy engagement is also urgently needed to ensure that decision-makers always rely on robust data to design and implement reproductive health and other policies.

Several studies have shown how even in highly legally restricted countries, women can be supported with information on what to do about an unplanned pregnancy, where to seek support, and how to arrive at a safe decision [76, 79]. The internet and hotlines can be a resource for many women, regardless of their social network and demographics, that increases knowledge of safe methods, sources, and what to expect. Our review shows the internet is burgeoning space for consultation and action. Formal harm reduction programs – including internet-based telemedicine, hotlines, and accompaniment models – that present safe abortion options in legally restrictive settings have had success supporting women to terminate safely [14, 76–86]. However, existing laws constrain such programmatic efforts; thus, program managers need to exercise caution to limit program’s exposure to legal or other repercussions. One such way is to work closely with local stakeholders and civil society organizations to ensure buy in and support for these harm reduction efforts.

Our findings underscore that many women still know about and use a range of unsafe methods to terminate an unintended pregnancy; thus, even in highly restrictive settings, quality PAC must be available for treatment of abortion-related complications. Although medical abortion is gaining popularity and may meet some women’s preferences for what they deem most ‘safe’ (socially), some women will continue to prefer surgical abortion for a variety of reasons, even with all safety considerations being equal.

Although this review unearthed several important findings, gaps remain – especially as we reflect on the Coast et al*.*[24] framework, such as how previous abortion experiences and women’s autonomy and self-efficacy influence decision-making. Available literature used as part of this review only superficially addressed women’s disclosure experiences, especially related to negotiation during the decision-making process, which suggests this is an area of further inquiry. We reviewed little information about power imbalances within relationships and gender norms facilitate or inhibit women’s ability to enact their abortion preferences. In addition, the literature in this review did not untangle how non-linear trajectories impact decision-making on how, when, and where to abort. More research is needed to fully understand the many factors that influence women’s abortion decision-making in highly legally restrictive settings, and to make comprehensive programmatic and policy recommendations. Our findings also highlight the need for more research on whether and how interventions to promote access to safe abortion in highly restrictive legal contexts are addressing the decision-making trajectories of women seeking abortion and promoting access to services that are both medically and socially safe for women.

We conducted our review within the bounds of the search criteria and the assets of the team, which constrained the geographic, linguistic, and timeframe scope. This is especially true for articles in French and Portuguese, as many of the countries included in this review speak those language predominantly. Relatedly, included studies concentrated in only a few of the many countries in Latin America and Africa so findings and recommendations may not be representative of or relevant to the entirety of those regions. We also restricted our search to peer-reviewed articles. As such, we may have excluded pertinent literature, including studies published in the grey literature or in French. Other decisions we made to focus the scope of our review, such as including only articles presenting data from women with first-hand experience, meant some rich, seminal articles about the decisions of how, when and where to abort were excluded (e.g. those showcasing providers’ perspectives). Included literature is likely limited to individuals identifying as women, and as such does not reflect the experiences of individuals who may not identify as women, to which the issue of abortion decision-making may still apply. Guttamcher’s 2017 country categorization of legality [23] is one of several tools available for understanding the legal context of abortion globally, and our use of it means we included countries with vastly differing legal contexts into this review. Many of the same factors influencing the decision to abort are the same as those influencing when, where and how; as such we may not have full distinction between the two decision-making processes in our synthesis.

The abortion decision-making process is complex. We focused on articles that explicitly discussed the choices and decisions that individuals made in varying contexts; most often, these were decisions that women were aware they were making. However, women make implicit decisions, in these cases often forced on them by circumstance – be that poverty, option scarcity, or within limited social networks. Our review does not cover passive or implicit decision making nor an investigation of the cognitive aspects related to decision-making that might be at play in one’s abortion trajectory.

Despite these limitations, our review contributes knowledge on decisions that women make related to abortion care-seeking *after* they have made the decision to terminate in a legally restrictive setting. This is a priority research area as the decisions a woman makes during this time determine whether she obtains a safe abortion, can exercise her human right to bodily autonomy, and impacts the likelihood of experience related injuries or even death. We used rigorous and comprehensive search methods involving 10 databases and employed a thorough article screening process involving two reviewers. Diversity on our team allowed us to include articles in Spanish, in addition to English, and represent research across diverse settings.

## Conclusion

Women’s decision-making process related to how, when and where to terminate a pregnancy in SSA and LAC countries with highly restrictive abortion laws is complex and shaped by myriad factors. This review provides important insight regarding what influences women’s termination trajectories and their impact on the safety of women’s abortions and their ability to decide their future. Understanding what aspects of available abortion options, or lack thereof, women prioritize in their decision-making process can enable stakeholders to better meet women’s abortion needs to the full limit of the law and maximize access to safer options within a harm reduction framework for those abortions obtained outside legal indications. More research is needed to understand these factors and make comprehensive policy and programmatic recommendations in legally restrictive settings.

## Supplementary Information

**Additional file 1.** Search terms.

## Data Availability

Data sharing is not applicable to this article as no datasets were generated or analyzed during the current study. All data used in this paper has been published elsewhere, and the full list of references is available in the paper.

## Footnotes

1 We used Guttmacher’s ‘*Abortion Legality Worldwide’ map and their definition of any country scoring between 1 and 4 as ‘highly legally restrictive*’[86]. We did not include countries ranked as a 5 or 6, which related to countries where abortion was defined as broadly legal.
