# Supplementary material for: How, when and where? A systematic review on abortion decision making in legally restricted settings in sub-Saharan Africa, Latin America, and the Caribbean
Source: BMC Womens Health. 2022 Oct 10;22:415. doi: 10.1186/s12905-022-01962-0 (PMC9552475; doi:10.1186/s12905-022-01962-0)
Supplement: Supplementary file 1 — Additional file 1. Search terms. [file 12905_2022_1962_MOESM1_ESM.docx]

**Supplemental Document 1: Search Terms for Review on Factors Influencing Women’s Decision Making in Obtaining an Induced Abortion**

| **Database** | **First hits** | **From 2020** | **Difference** | **Search Update dates** |
| --- | --- | --- | --- | --- |
| PubMed | 1,862 | 2,734 | 872 | June 19, 2019-- |
| Embase | 3,086 | 4,058 | 972 | June 19, 2019 |
| Web of Science | 1,975 | 2,791 | 816 | June 19, 2019 |
| *POPLINE* | *1,259* |  |  | *n/a No longer available* |
| CINAHL | 760 | 1,198 | 438 | June 21, 2019 |
| Global Health (Ovid) | 1,444 | 1,811 | 367 | June 24, 2019 |
| WHO Regional Databases | 695 | 1,039 | 344 | June 21, 2019 |
| Cochrane trials | 140 | 182 | 42 | June 24, 2019 |
| Cochrane reviews | 365 | 443 | 78 | June 24, 2019 |
| Cochrane protocols | 34 | 32 | 0-32 | June 24, 2019 |

**Search Structure:** Women’s Experience AND Abortion AND Geographic area

**Time Range:** January 1, 2000 and later

Update run 5/19/2022 by rerunning full searches, deduplicating against existing records.

**PubMed Search**

***1. Women’s Experience/Decision Making:***

(women's experience*[tw] OR women's perspective*[tw] OR

decision making[mesh] OR choice behavior[mesh] OR decision*[tw] OR choice*[tw] OR choosing[tw] OR choose[tw] OR influence*[tw] OR factor*[tw] OR

"Friends"[Mesh] OR "Interpersonal Relations"[Mesh] OR "Spouses"[Mesh] OR "Family"[Mesh] OR friend*[tw] OR peer[tw] OR peers[tw] OR partner[tw] OR partners[tw] OR husband*[tw] OR spouse*[tw] OR boyfriend*[tw] OR famil*[tw] OR mother in law[tw] OR extended famil*[tw] OR social network*[tw] OR

"Self Efficacy"[Mesh] OR self efficacy[tw] OR agency[ti] OR

health knowledge, attitudes, practice[mesh] OR "Social Stigma"[Mesh] OR "Shame"[Mesh] OR health knowledge[tw] OR (attitude*[tw] AND health[tw]) OR shame[tw] OR stigma[tw])

***2. Abortion:***

("Abortion, Induced"[Mesh] OR "Abortion, Criminal"[Mesh] OR abortion*[tw] OR (pregnancy[tw] AND terminat*[tw]) OR (menstr*[tw] AND regulation [tw]) OR (period[tw] AND regulation[tw]) )

***3. Geography:***

(“sub-Saharan Africa”[MeSH] OR “sub-Saharan Africa”[tw] OR sub-Sahara[tw] OR subsahara[tw] OR “west Africa”[tw] OR “east Africa”[tw] OR “western Africa”[tw] OR “eastern africa”[tw] OR “central Africa”[tw] OR “southern Africa”[tw] OR “Cameroon” [MeSH] OR “Central African Republic” [MeSH] OR “Chad” [MeSH] OR “Congo” [MeSH] OR “Democratic Republic of the Congo” [MeSH] OR “Equatorial Guinea” [MeSH] OR “Gabon” [MeSH] OR “Sao Tome and Principe” [MeSH] OR “Burundi” [MeSH] OR “Djibouti” [MeSH] OR “Eritrea” [MeSH] OR “Ethiopia” [MeSH] OR “Kenya” [MeSH] OR “Rwanda” [MeSH] OR “Somalia” [MeSH] OR “South Sudan” [MeSH] OR “Sudan” [MeSH] OR “Tanzania” [MeSH] OR “Uganda” [MeSH] OR “Angola” [MeSH] OR “Botswana” [MeSH] OR “Lesotho” [MeSH] OR “Malawi” [MeSH] OR “Mozambique” [MeSH] OR “Namibia” [MeSH] OR “Swaziland” [MeSH] OR “Zimbabwe” [MeSH] OR “Benin” [MeSH] OR “Burkina Faso” [MeSH] OR “Cote d'Ivoire” [MeSH] OR “Gambia” [MeSH] OR “Ghana” [MeSH] OR “Guinea” [MeSH] OR “Guinea-Bissau” [MeSH] OR “Liberia” [MeSH] OR “Mali” [MeSH] OR “Mauritania” [MeSH] OR “Niger” [MeSH] OR “Nigeria” [MeSH] OR “Senegal” [MeSH] OR “Sierra Leone” [MeSH] OR “Togo” [MeSH] OR Argentina [MeSH] OR Bolivia [MeSH] OR Brazil [MeSH] OR Chile [MeSH] OR Colombia [MeSH] OR Ecuador [MeSH] OR “French Guiana” [MeSH] OR Paraguay [MeSH] OR Peru [MeSH] OR Suriname [MeSH] OR Uruguay[MeSH] OR Venezuela [MeSH] OR Aruba [MeSH] OR Curacao [MeSH] OR “Sint Maarten”[MeSH] OR West Indies [MeSH] OR “Antigua and Barbuda” [MeSH] OR Bahamas [MeSH] OR Dominica [MeSH] OR “Dominican Republic” [MeSH] OR Grenada [MeSH] OR Guadeloupe [MeSH] OR Haiti [MeSH] OR Jamaica [MeSH] OR Martinique [MeSH] OR “Saint Kitts and Nevis” [MeSH] OR “Saint Lucia” [MeSH] OR “Trinidad and Tobago” [MeSH] OR “Costa Rica” [MeSH] OR “El Salvador” [MeSH] OR Guatemala [MeSH] OR Honduras [MeSH] OR Nicaragua [MeSH] OR Panama [MeSH] OR “Latin America” [MeSH] OR “Cameroon” [tw] OR “Central African Republic” [tw] OR “Chad” [tw] OR “Congo” [tw] OR “Democratic Republic of the Congo” [tw] OR “Equatorial Guinea” [tw] OR “Gabon” [tw] OR “Sao Tome and Principe” [tw] OR “Burundi” [tw] OR “Djibouti” [tw] OR “Eritrea” [tw] OR “Ethiopia” [tw] OR “Kenya” [tw] OR “Rwanda” [tw] OR “Somalia” [tw] OR “South Sudan” [tw] OR “Sudan” [tw] OR “Tanzania” [tw] OR “Uganda” [tw] OR “Angola” [tw] OR “Botswana” [tw] OR “Lesotho” [tw] OR “Malawi” [tw] OR “Mozambique” [tw] OR “Namibia” [tw] OR “Swaziland” [tw] OR “Zimbabwe” [tw] OR “Benin” [tw] OR “Burkina Faso” [tw] OR “Cote d'Ivoire” [tw] OR “Gambia” [tw] OR “Ghana” [tw] OR “Guinea” [tw] OR “Guinea-Bissau” [tw] OR “Liberia” [tw] OR “Mali” [tw] OR “Mauritania” [tw] OR “Niger” [tw] OR “Nigeria” [tw] OR “Senegal” [tw] OR “Sierra Leone” [tw] OR “Togo” [tw] OR Argentina [tw] OR Bolivia [tw] OR Brazil [tw] OR Chile [tw] OR Colombia [tw] OR Ecuador [tw] OR “French Guiana” [tw] OR Paraguay [tw] OR Peru [tw] OR Suriname [tw] OR Venezuela [tw] OR Aruba [tw] OR Curacao [tw] OR “Sint Maarten”[tw] OR “St. Maarten” [tw] OR “saint Maarten”[tw] OR West Indies [tw] OR “Antigua and Barbuda” [tw] OR Bahamas [tw] OR Dominica [tw] OR “Dominican Republic” [tw] OR Grenada [tw] OR Guadeloupe [tw] OR Haiti [tw] OR Jamaica [tw] OR Martinique [tw] OR “Saint Kitts and Nevis” [tw] OR “Saint Lucia” [tw] OR “Trinidad and Tobago” [tw] OR “Costa Rica” [tw] OR “El Salvador” [tw] OR Guatemala [tw] OR Honduras [tw] OR Nicaragua [tw] OR Panama [tw] OR “Latin America” [tw] OR Uruguay[tw])

**Embase**

(('women* experience*':ab,kw,ti,jt,lnk OR 'women* perspective*':ab,kw,ti,jt,lnk) OR ('decision making'/exp OR (decision*:ab,kw,ti,lnk OR choice*:ab,kw,ti,lnk OR choosing:ab,kw,ti,lnk OR choose:ab,kw,ti,lnk OR influence*:ab,kw,ti,lnk OR factor*:ab,kw,ti,lnk)) OR (('friend'/exp OR 'human relation'/exp OR 'spouse'/exp OR 'family'/exp) OR (friend*:ab,kw,ti,jt,lnk OR peer:ab,kw,ti,jt,lnk OR peers:ab,kw,ti,jt,lnk OR partner:ab,kw,ti,jt,lnk OR partners:ab,kw,ti,jt,lnk OR husband*:ab,kw,ti,jt,lnk OR spouse*:ab,kw,ti,jt,lnk OR boyfriend*:ab,kw,ti,jt,lnk OR famil*:ab,kw,ti,jt,lnk OR 'mother in law':ab,kw,ti,jt,lnk OR 'extended famil*':ab,kw,ti,jt,lnk OR 'social network*':ab,kw,ti,jt,lnk)) OR ('self concept'/exp OR ('self-efficacy':ab,kw,ti,jt,lnk OR agency:ti)) OR (('attitude to health'/exp OR 'social stigma'/exp) OR (shame:ab,kw,ti,jt,lnk OR 'health knowledge':ab,kw,ti,jt,lnk OR ((attitude* NEAR/3 health):ab,kw,ti,jt,lnk) OR stigma:ab,kw,ti,jt,lnk))))

AND

(('induced abortion'/exp OR 'illegal abortion'/exp) OR (abortion*:ab,kw,ti,lnk OR ((pregnancy NEAR/3 terminat*):ab,kw,ti,lnk) OR ((menstr* NEAR/3 regulation):ab,kw,ti,lnk) OR ((period NEAR/3 regulation):ab,kw,ti,lnk)) OR 'pregnancy termination'/de)

AND

(('antigua and barbuda'/exp OR 'antigua and barbuda' OR 'burkina faso'/exp OR 'burkina faso' OR 'central africa'/exp OR 'central africa' OR 'central african republic'/exp OR 'central african republic' OR 'costa rica'/exp OR 'costa rica' OR 'cote d ivoire'/exp OR 'cote d ivoire' OR 'ivory coast'/exp OR 'ivory coast' OR 'dominican republic'/exp OR 'dominican republic' OR 'east africa'/exp OR 'east africa' OR 'eastern africa'/exp OR 'eastern africa' OR 'el salvador'/exp OR 'el salvador' OR 'equatorial guinea'/exp OR 'equatorial guinea' OR 'french guiana'/exp OR 'french guiana' OR 'guinea-bissau'/exp OR 'guinea-bissau' OR 'latin america'/exp OR 'latin america' OR 'saint kitts and nevis'/exp OR 'saint kitts and nevis' OR 'saint lucia'/exp OR 'saint lucia' OR 'saint maarten' OR 'sao tome and principe'/exp OR 'sao tome and principe' OR 'sierra leone'/exp OR 'sierra leone' OR 'sint maarten'/exp OR 'sint maarten' OR 'south sudan'/exp OR 'south sudan' OR 'southern africa'/exp OR 'southern africa' OR 'st. maarten'/exp OR 'st. maarten' OR 'africa south of the sahara'/exp OR 'sub-saharan africa' OR 'trinidad and tobago'/exp OR 'trinidad and tobago' OR 'west africa'/exp OR 'west africa' OR 'western africa'/exp OR 'western africa' OR 'angola'/exp OR angola OR 'argentina'/exp OR argentina OR 'aruba'/exp OR aruba OR 'bahamas'/exp OR bahamas OR 'benin'/exp OR benin OR 'bolivia'/exp OR bolivia OR 'botswana'/exp OR botswana OR 'brazil'/exp OR brazil OR 'burundi'/exp OR burundi OR 'cameroon'/exp OR cameroon OR 'chad'/exp OR chad OR 'chile'/exp OR chile OR 'colombia'/exp OR colombia OR 'congo'/exp OR congo OR 'curacao'/exp OR curacao OR 'djibouti'/exp OR djibouti OR 'dominica'/exp OR dominica OR 'ecuador'/exp OR ecuador OR 'eritrea'/exp OR eritrea OR 'ethiopia'/exp OR ethiopia OR 'gabon'/exp OR gabon OR 'gambia'/exp OR gambia OR 'grenada'/exp OR grenada OR 'guadeloupe'/exp OR guadeloupe OR 'guatemala'/exp OR guatemala OR 'guinea'/exp OR guinea OR 'haiti'/exp OR haiti OR 'honduras'/exp OR honduras OR 'jamaica'/exp OR jamaica OR 'kenya'/exp OR kenya OR 'lesotho'/exp OR lesotho OR 'liberia'/exp OR liberia OR 'malawi'/exp OR malawi OR 'mali'/exp OR mali OR 'martinique'/exp OR martinique OR 'mauritania'/exp OR mauritania OR 'mozambique'/exp OR mozambique OR 'namibia'/exp OR namibia OR 'nicaragua'/exp OR nicaragua OR 'niger'/exp OR niger OR 'nigeria'/exp OR nigeria OR 'panama'/exp OR panama OR 'paraguay'/exp OR paraguay OR 'peru'/exp OR peru OR 'rwanda'/exp OR rwanda OR 'senegal'/exp OR senegal OR 'somalia'/exp OR somalia OR subsahara OR 'sub sahara' OR 'sudan'/exp OR sudan OR 'suriname'/exp OR suriname OR eswatini/exp OR eswatini OR swaziland OR 'tanzania'/exp OR tanzania OR 'togo'/exp OR togo OR 'uganda'/exp OR uganda OR 'uruguay'/exp OR uruguay OR 'venezuela'/exp OR venezuela OR 'west indies'/exp OR 'west indies' OR 'zimbabwe'/exp OR zimbabwe)

AND

(2000:py OR 2001:py OR 2002:py OR 2003:py OR 2004:py OR 2005:py OR 2006:py OR 2007:py OR 2008:py OR 2009:py OR 2010:py OR 2011:py OR 2012:py OR 2013:py OR 2014:py OR 2015:py OR 2016:py OR 2017:py OR 2018:py OR 2019:py OR 2020:py OR 2021:py OR 2022:py)

**Web of Science**

TS=(women's experience* OR women's perspective* OR decision* OR choice* OR choosing OR choose OR influence* OR factor* OR "Interpersonal Relations" OR "Spouses" OR Family OR friend* OR peer OR peers OR partner OR partners OR husband* OR spouse* OR boyfriend* OR famil* OR “mother in law” OR “extended famil*” OR “social network*” OR Self-efficacy OR agency OR “health knowledge” OR (attitude* AND health) OR shame OR stigma)

AND

TS=(abortion* OR (pregnancy AND terminat*) OR (menstr* AND regulation ) OR (period AND regulation) )

AND

TS=(“Antigua and Barbuda” OR “Burkina Faso” OR “Central Africa” OR “Central African Republic” OR “Costa Rica” OR “Cote d'Ivoire” OR “Dominican Republic” OR “East Africa” OR “Eastern Africa” OR “El Salvador” OR “Equatorial Guinea” OR “French Guiana” OR “Guinea-Bissau” OR “Latin America” OR “Saint Kitts and Nevis” OR “Saint Lucia” OR “saint Maarten” OR “Sao Tome and Principe” OR “Sierra Leone” OR “Sint Maarten” OR “South Sudan” OR “southern Africa” OR “St. Maarten” OR “Sub-Saharan Africa” OR “Trinidad and Tobago” OR “West Africa” OR “Western Africa” OR Angola OR Argentina OR Aruba OR Bahamas OR Benin OR Bolivia OR Botswana OR Brazil OR Burundi OR Cameroon OR Chad OR Chile OR Colombia OR Congo OR Curacao OR Djibouti OR Dominica OR Ecuador OR Eritrea OR Ethiopia OR Eswatini OR Gabon OR Gambia OR Grenada OR Guadeloupe OR Guatemala OR Guinea OR Haiti OR Honduras OR Jamaica OR Kenya OR Lesotho OR Liberia OR Malawi OR Mali OR Martinique OR Mauritania OR Mozambique OR Namibia OR Nicaragua OR Niger OR Nigeria OR Panama OR Paraguay OR Peru OR Rwanda OR Senegal OR Somalia OR subsahara OR sub-Sahara OR Sudan OR Suriname OR Swaziland OR Tanzania OR Togo OR Uganda OR Uruguay OR Venezuela OR West Indies OR Zimbabwe)

**POPLINE** [**https://www.popline.org/**](https://www.popline.org/)

("women's experience" OR "women's experiences" OR "women's perspective" OR "women's perspectives" OR decision* OR choice* OR choosing OR choose OR influence* OR factor* OR "Interpersonal Relations" OR Spouses OR Family OR friend* OR peer OR peers OR partner OR partners OR husband* OR spouse* OR boyfriend* OR famil* OR "mother in law" OR "extended family" OR "extended families" OR "social network" OR "social networks" OR "social networking" OR Self-efficacy OR agency OR "health knowledge" OR (attitude* AND health) OR shame OR stigma)

AND

(abortion* OR (pregnancy AND terminat*) OR (menstr* AND regulation ) OR (period AND regulation) )

AND

("Antigua and Barbuda" OR "Burkina Faso" OR "Central Africa" OR "Central African Republic" OR "Costa Rica" OR "Cote d'Ivoire" OR "Dominican Republic" OR "East Africa" OR "Eastern Africa" OR "El Salvador" OR "Equatorial Guinea" OR "French Guiana" OR "Guinea-Bissau" OR "Latin America" OR "Saint Kitts and Nevis" OR "Saint Lucia" OR "saint Maarten" OR "Sao Tome and Principe" OR "Sierra Leone" OR "Sint Maarten" OR "South Sudan" OR "southern Africa" OR "St. Maarten" OR "Sub-Saharan Africa" OR "Trinidad and Tobago" OR "West Africa" OR "Western Africa" OR Angola OR Argentina OR Aruba OR Bahamas OR Benin OR Bolivia OR Botswana OR Brazil OR Burundi OR Cameroon OR Chad OR Chile OR Colombia OR Congo OR Curacao OR Djibouti OR Dominica OR Ecuador OR Eritrea OR Ethiopia OR Gabon OR Gambia OR Grenada OR Guadeloupe OR Guatemala OR Guinea OR Haiti OR Honduras OR Jamaica OR Kenya OR Lesotho OR Liberia OR Malawi OR Mali OR Martinique OR Mauritania OR Mozambique OR Namibia OR Nicaragua OR Niger OR Nigeria OR Panama OR Paraguay OR Peru OR Rwanda OR Senegal OR Somalia OR subsahara OR sub-Sahara OR Sudan OR Suriname OR Swaziland OR Tanzania OR Togo OR Uganda OR Uruguay OR Venezuela OR "West Indies" OR Zimbabwe)

**Searching Notes:**

Limited to “added to POPLINE from January 1, 2000 to June 19, 2019”

Limited to English and Spanish

Limited to “journal articles” to exclude grey literature

Notes: POPLINE cannot process * inside quotes so the variations have been spelled out. Also, POPLINE cannot handle “smart quotes” like the ones around that phrase, i.e. curly quotes. IF you edit this search be sure that all single and double quotes are straight up and down. Safest to edit in a text editor.

**CINAHL**

**Platform: CINAHL Plus with full text**

| **#** | **Query** | **Limiters/Expanders** | **Results** |
| --- | --- | --- | --- |
| S11 | S3 AND S7 AND S10 | Limiters - Published Date: 20000101-20220531; Language: English, Spanish  Expanders - Apply related words; Apply equivalent subjects  Search modes - Boolean/Phrase | 1,198 |
| S10 | S8 OR S9 | Expanders - Apply related words; Apply equivalent subjects  Search modes - Boolean/Phrase | 2,633,903 |
| S9 | (women's experience* OR women's perspective* OR decision* OR choice* OR choosing OR choose OR influence* OR factor* OR "Interpersonal Relations" OR "Spouses" OR Family OR friend* OR peer OR peers OR partner OR partners OR husband* OR spouse* OR boyfriend* OR famil* OR “mother in law” OR “extended famil*” OR “social network*” OR Self-efficacy OR agency OR “health knowledge” OR (attitude* AND health) OR shame OR stigma) | Search modes - Boolean/Phrase | 2,620,937 |
| S8 | (MH "Decision Making") OR (MH "Friendship") OR (MH "Dating") OR (MH "Interpersonal Relations") OR (MH "Spouses") OR (MH "Significant Other") OR (MH "Family") OR (MH "Extended Family") OR (MH "Self-Efficacy") OR (MH "Health Knowledge") OR (MH "Attitude to Health") OR (MH "Stigma") OR (MH "Shame") | Search modes - Boolean/Phrase | 283,752 |
| S7 | S4 OR S5 OR S6 | Search modes - Boolean/Phrase | 171,843 |
| S6 | ("Antigua and Barbuda" OR "Burkina Faso" OR "Central Africa" OR "Central African Republic" OR "Costa Rica" OR "Cote d'Ivoire" OR "Dominican Republic" OR "East Africa" OR "Eastern Africa" OR "El Salvador" OR "Equatorial Guinea" OR "French Guiana" OR "Guinea-Bissau" OR "Latin America" OR "Saint Kitts and Nevis" OR "Saint Lucia" OR "saint Maarten" OR "Sao Tome and Principe" OR "Sierra Leone" OR "Sint Maarten" OR "South Sudan" OR "southern Africa" OR "St. Maarten" OR "Sub-Saharan Africa" OR "Trinidad and Tobago" OR "West Africa" OR "Western Africa" OR Angola OR Argentina OR Aruba OR Bahamas OR Benin OR Bolivia OR Botswana OR Brazil OR Burundi OR Cameroon OR Chad OR Chile OR Colombia OR Congo OR Curacao OR Djibouti OR Dominica OR Ecuador OR Eritrea OR Eswatini OR Ethiopia OR Gabon OR Gambia OR Grenada OR Guadeloupe OR Guatemala OR Guinea OR Haiti OR Honduras OR Jamaica OR Kenya OR Lesotho OR Liberia OR Malawi OR Mali OR Martinique OR Mauritania OR Mozambique OR Namibia OR Nicaragua OR Niger OR Nigeria OR Panama OR Paraguay OR Peru OR Rwanda OR Senegal OR Somalia OR subsahara OR sub-Sahara OR Sudan OR Suriname OR Swaziland OR Tanzania OR Togo OR Uganda OR Uruguay OR Venezuela OR "West Indies" OR Zimbabwe) | Search modes - Boolean/Phrase | 166,373 |
| S5 | (MH "Latin America") OR (MH "Uruguay") OR (MH "Suriname") OR (MH "Peru") OR (MH "Paraguay") OR (MH "French Guiana") OR (MH "Argentina") OR (MH "Bolivia") OR (MH "Brazil") OR (MH "Chile") OR (MH "Colombia") OR (MH "Ecuador") OR (MH "Venezuela") OR (MH "Costa Rica") OR (MH "El Salvador") OR (MH "Guatemala") OR (MH "Honduras") OR (MH "Nicaragua") OR (MH "Panama") OR (MH "Bahamas") OR (MH "Trinidad and Tobago") OR (MH "Dominican Republic") OR (MH "Dominica") OR (MH "Antigua") OR (MH "Haiti") OR (MH "Jamaica") OR (MH "Martinique") | Search modes - Boolean/Phrase | 77,041 |
| S4 | (MH "Africa South of the Sahara") OR (MH "Cameroon") OR (MH "Africa, Eastern") OR (MH "Africa, Southern") OR (MH "Africa, Western") OR (MH "Central African Republic") OR (MH "Burkina Faso") OR (MH "Cote d'Ivoire") OR (MH "Equatorial Guinea") OR (MH "Democratic Republic of the Congo") OR (MH "Guinea-Bissau") OR (MH "Sierra Leone") OR (MH "Sudan") OR (MH "Angola") OR (MH "Botswana") OR (MH "Benin") OR (MH "Burundi") OR (MH "Chad") OR (MH "Eritrea") OR (MH "Ethiopia") OR (MH "Gabon") OR (MH "Djibouti") OR (MH "Gambia") OR (MH "Ghana") OR (MH "Guinea") OR (MH "Kenya") OR (MH "Lesotho") OR (MH "Liberia") OR (MH "Mali") OR (MH "Malawi") OR (MH "Mozambique") OR (MH "Mauritania") OR (MH "Namibia") OR (MH "Niger") OR (MH "Nigeria") OR (MH "Senegal") OR (MH "Rwanda") OR (MH "Somalia") OR (MH "Eswatini") OR (MH "Tanzania") OR (MH "Togo") OR (MH "Zimbabwe") OR (MH "Uganda") | Search modes - Boolean/Phrase | 56,552 |
| S3 | S1 OR S2 | Search modes - Boolean/Phrase | Display |
| S2 | (pregnancy AND terminat*) OR (menstr* AND regulat*) OR (period AND regulat*) | Search modes - Boolean/Phrase | Display |
| S1 | (MH "Abortion, Criminal") OR (MH "Abortion, Induced") OR "abortion" | Search modes - Boolean/Phrase | Display |

**Searching Notes:**

Used Limits for English and Spanish (Or’ed two sets together)

Not all countries had unique subject headings

For update: selected both English and Spanish simultaneously from drop down list.

**Global Health (Ovid)**

Global Health <1910 to 2022 Week 19>

1 ("women's experiences" or "women's experience" or "women's perspective" or "women's perspectives").mp. [mp=abstract, title, original title, broad terms, heading words, identifiers, cabicodes] 1116

2 decision making/ 11379

3 (decision* or choice* or choos* or influenc* or factor*).mp. [mp=abstract, title, original title, broad terms, heading words, identifiers, cabicodes] 1149330

4 interpersonal relations/ 3074

5 exp families/ or extended families/ 19424

6 (friend* or peer or peers or partner* or husband* or spous* or boyfriend* or famil* or mother-in-law* or social network*).mp. [mp=abstract, title, original title, broad terms, heading words, identifiers, cabicodes] 296740

7 (self-efficacy or agency).mp. [mp=abstract, title, original title, broad terms, heading words, identifiers, cabicodes] 23329

8 (health education or health promotion or attitudes).sh. 113147

9 social stigma/ 5737

10 ("health knowledge" or (attitude* and health) or shame or stigma).mp. [mp=abstract, title, original title, broad terms, heading words, identifiers, cabicodes] 69238

11 1 or 2 or 3 or 4 or 5 or 6 or 7 or 8 or 9 or 10 1398068

12 abortion.mp. [mp=abstract, title, original title, broad terms, heading words, identifiers, cabicodes] 15976

13 induced abortion/ 2047

14 ((pregnancy and terminat*) or (menstr* and regulation) or (period and regulation)).mp. [mp=abstract, title, original title, broad terms, heading words, identifiers, cabicodes] 7017

15 12 or 13 or 14 21843

16 exp caribbean/ 24842

17 exp "africa south of sahara"/ or exp central africa/ or exp east africa/ or exp southern africa/ or exp west africa/ 244330

18 exp Central America/ 15298

19 exp South America/ 170916

20 ("Antigua and Barbuda" or "Burkina Faso" or "Central Africa" or "Central African Republic" or "Costa Rica" or "Cote d'Ivoire" or "Dominican Republic" or "East Africa" or "Eastern Africa" or "El Salvador" or "Equatorial Guinea" or "French Guiana" or "Guinea-Bissau" or "Latin America" or "Saint Kitts and Nevis" or "Saint Lucia" or "saint Maarten" or "Sao Tome and Principe" or "Sierra Leone" or "Sint Maarten" or "South Sudan" or "southern Africa" or "St. Maarten" or "Sub-Saharan Africa" or "Trinidad and Tobago" or "West Africa" or "Western Africa" or Angola or Argentina or Aruba or Bahamas or Benin or Bolivia or Botswana or Brazil or Burundi or Cameroon or Chad or Chile or Colombia or Congo or Curacao or Djibouti or Dominica or Ecuador or Eritrea or Eswatini or Ethiopia or Gabon or Gambia or Grenada or Guadeloupe or Guatemala or Guinea or Haiti or Honduras or Jamaica or Kenya or Lesotho or Liberia or Malawi or Mali or Martinique or Mauritania or Mozambique or Namibia or Nicaragua or Niger or Nigeria or Panama or Paraguay or Peru or Rwanda or Senegal or Somalia or subsahara or sub-Sahara or Sudan or Suriname or Swaziland or Tanzania or Togo or Uganda or Uruguay or Venezuela or "West Indies" or Zimbabwe).mp. [mp=abstract, title, original title, broad terms, heading words, identifiers, cabicodes] 530735

21 16 or 17 or 18 or 19 or 20 536627

22 11 and 15 and 21 2260

23 11 and 15 and 21 2260

24 limit 22 to yr="2000 - 2022" 1997

25 limit 24 to (english or spanish) 1811

**Searching Notes:**

Did not explode “decision making” as narrower terms included “organizational decision making” and other non-relevant terms.

For update: used publication years and “English” and “Spanish” limits.

**WHO Regional Databases** [**http://pesquisa.bvsalud.org/portal/advanced/?lang=en**](http://pesquisa.bvsalud.org/portal/advanced/?lang=en)

((("women's experience" OR "women's experiences" OR "women's perspective" OR "women's perspectives" OR decision* OR choice* OR choosing OR choose OR influence* OR factor* OR "Interpersonal Relations" OR Spouses OR Family OR friend* OR peer OR peers OR partner OR partners OR husband* OR spouse* OR boyfriend* OR famil* OR "mother in law" OR "extended family" OR "extended families" OR "social network" OR "social networks" OR "social networking" OR Self-efficacy OR agency OR "health knowledge" OR (attitude* AND health) OR shame OR stigma) ))

AND

(tw:((abortion* OR (pregnancy AND terminat*) OR (menstr* AND regulation ) OR (period AND regulation) )))

AND

(tw:(("Antigua and Barbuda" OR "Burkina Faso" OR "Central Africa" OR "Central African Republic" OR "Costa Rica" OR "Cote d'Ivoire" OR "Dominican Republic" OR "East Africa" OR "Eastern Africa" OR "El Salvador" OR "Equatorial Guinea" OR "French Guiana" OR "Guinea-Bissau" OR "Latin America" OR "Saint Kitts and Nevis" OR "Saint Lucia" OR "saint Maarten" OR "Sao Tome and Principe" OR "Sierra Leone" OR "Sint Maarten" OR "South Sudan" OR "southern Africa" OR "St. Maarten" OR "Sub-Saharan Africa" OR "Trinidad and Tobago" OR "West Africa" OR "Western Africa" OR Angola OR Argentina OR Aruba OR Bahamas OR Benin OR Bolivia OR Botswana OR Brazil OR Burundi OR Cameroon OR Chad OR Chile OR Colombia OR Congo OR Curacao OR Djibouti OR Dominica OR Ecuador OR Eritrea OR Eswatini OR Ethiopia OR Gabon OR Gambia OR Grenada OR Guadeloupe OR Guatemala OR Guinea OR Haiti OR Honduras OR Jamaica OR Kenya OR Lesotho OR Liberia OR Malawi OR Mali OR Martinique OR Mauritania OR Mozambique OR Namibia OR Nicaragua OR Niger OR Nigeria OR Panama OR Paraguay OR Peru OR Rwanda OR Senegal OR Somalia OR subsahara OR sub-Sahara OR Sudan OR Suriname OR Swaziland OR Tanzania OR Togo OR Uganda OR Uruguay OR Venezuela OR "West Indies" OR Zimbabwe) ))

**Searching Notes:**

Excluded Medline

Limited to Spanish and English.

Did NOT limit to Humans, as indexing appeared incomplete

Limited to Date of publication by importing all and removing those with explicit years less than 2000

Update: followed procedure, however database set became: LILACS, BINACIS, MedCarib, PAHO, BDENF-Nursing, PAHO-IRIS, LIPECS

**Cochrane Library** (Includes Cochrane Reviews, Cochrane Protocols, and Trials)

ID Search Hits

#1 "women's experiences" OR "women's experience" OR "women's perspective" OR "women's perspectives" 641

#2 MeSH descriptor: [Decision Making] explode all trees 3864

#3 MeSH descriptor: [Choice Behavior] explode all trees 1384

#4 decision* OR choice* OR choos* OR influenc* OR factor* 340727

#5 MeSH descriptor: [Friends] explode all trees 126

#6 MeSH descriptor: [Interpersonal Relations] explode all trees 5946

#7 MeSH descriptor: [Spouses] explode all trees 315

#8 MeSH descriptor: [Family] explode all trees 8407

#9 friend* OR peer OR peers OR partner* OR husband* OR spous* OR boyfriend* OR famil* OR mother-in-law* OR social network* 72650

#10 MeSH descriptor: [Self Efficacy] explode all trees 2797

#11 self-efficacy OR agency 14534

#12 MeSH descriptor: [Health Knowledge, Attitudes, Practice] explode all trees 5552

#13 “health knowledge” OR (attitude* AND health) OR shame OR stigma 19357

#14 MeSH descriptor: [Social Stigma] explode all trees 189

#15 MeSH descriptor: [Shame] explode all trees 57

#16 #1 OR #2 OR #3 OR #4 OR #5 OR #6 OR #7 OR #8 OR #9 OR #10 OR #11 OR #12 OR #13 OR #14 OR #15 403192

#17 MeSH descriptor: [Abortion, Induced] explode all trees 1027

#18 MeSH descriptor: [Abortion, Criminal] explode all trees 1

#19 abortion 5141

#20 (pregnancy AND terminat*) OR (menstr* AND regulation ) OR (period AND regulation) 6350

#21 #17 OR #18 OR #19 OR #20 10436

#22 MeSH descriptor: [Africa South of the Sahara] explode all trees 5778

#23 MeSH descriptor: [Latin America] explode all trees 110

#24 MeSH descriptor: [Caribbean Region] explode all trees 383

#25 MeSH descriptor: [Central America] explode all trees 257

#26 MeSH descriptor: [South America] explode all trees 2204

#27 ("Antigua and Barbuda" OR "Burkina Faso" OR "Central Africa" OR "Central African Republic" OR "Costa Rica" OR "Cote d'Ivoire" OR "Dominican Republic" OR "East Africa" OR "Eastern Africa" OR "El Salvador" OR "Equatorial Guinea" OR "French Guiana" OR "Guinea-Bissau" OR "Latin America" OR "Saint Kitts and Nevis" OR "Saint Lucia" OR "saint Maarten" OR "Sao Tome and Principe" OR "Sierra Leone" OR "Sint Maarten" OR "South Sudan" OR "southern Africa" OR "St. Maarten" OR "Sub-Saharan Africa" OR "Trinidad and Tobago" OR "West Africa" OR "Western Africa" OR Angola OR Argentina OR Aruba OR Bahamas OR Benin OR Bolivia OR Botswana OR Brazil OR Burundi OR Cameroon OR Chad OR Chile OR Colombia OR Congo OR Curacao OR Djibouti OR Dominica OR Ecuador OR Eritrea OR Ethiopia OR Gabon OR Gambia OR Grenada OR Guadeloupe OR Guatemala OR Guinea OR Haiti OR Honduras OR Jamaica OR Kenya OR Lesotho OR Liberia OR Malawi OR Mali OR Martinique OR Mauritania OR Mozambique OR Namibia OR Nicaragua OR Niger OR Nigeria OR Panama OR Paraguay OR Peru OR Rwanda OR Senegal OR Somalia OR subsahara OR sub-Sahara OR Sudan OR Suriname OR Swaziland OR Tanzania OR Togo OR Uganda OR Uruguay OR Venezuela OR "West Indies" OR Zimbabwe) 36463

#28 #22 OR #23 OR #24 OR #25 OR #26 OR #27 37922

#29 #16 AND #21 AND #28 555

#30 MeSH descriptor: [South Africa] explode all trees 982

#31 #29 NOT #30 544

**Searching Notes:**

Searches not limited by language.

Result total includes 1 editorial and 1 special collection, both not downloaded.

Date limits January 1, 2000+ applied only reduced the total Cochrane reviews by 3.

Cochrane Reviews, Protocols, and Trials added to main database.

Update: As above, however total results now include 1 editorial, 3 special collections, and 3 clinical answers, all not downloaded. Adding Eswatini to the country keywords did not change total results.
